# A Note about Crosslinking Density in Imprinting Polymerization

**DOI:** 10.3390/molecules26175139

**Published:** 2021-08-25

**Authors:** Anja Mueller

**Affiliations:** Department of Chemistry and Biochemistry, Central Michigan University, Mount Pleasant, MI 48859, USA; muell1a@cmich.edu; Tel.: +1-989-774-3956

**Keywords:** molecularly imprinted polymer, MIP, crosslinking density, specific binding

## Abstract

Imprinting polymerization is an exciting technique since it leads to specific binding sites, which are the basis of a variety of applications, such as sensors, detectors, and catalysts. The specific binding sites are created using templates and then fixing the structure of the binding site with crosslinking. The literature review of imprinting polymerizations shows that the crosslinking density governs the physical properties of the resulting molecularly imprinted polymer (MIP). It is also a factor governing the capacity and the selectivity of MIPs. Reviewing polymer science data and theory, the crosslinking density commonly used in MIP synthesis is unusually high. The data reviewed here suggest that more research is needed to determine the optimal crosslinking density for MIPs.

## 1. Introduction

Imprinting polymerization is an exciting technique: By just adding one additional step to the synthesis of a common polymer, a material can be made specific to a chemical. Basically, that chemical, the template, is added to the synthesis solution. The monomers will surround the template automatically and form the strongest bonds possible, since thermodynamically that happens to be the lowest energy state and thus is preferred. The monomers will then be polymerized and crosslinked, and with that the three dimensional structure with the strongest bonds to the template will be conserved. The additional step is to remove the template. This results in a pocket ideal for rebinding the template [1].

How useful specific binding is can be seen in biochemistry. A cell contains a large number of compounds and intermediates, but despite that, enzymes choose one specific compound to react without any side products, simply by providing a very specific binding site. In organic chemistry that is only possible in very few cases with complicated, many-step syntheses resulting in low yields. Another example are antibodies that recognize one specific compound on the surface of pathogenic bacteria to then destroy those bacteria and thus prevent a possible deadly infection. Imprinting polymerization promises specific binding to allow for analogous applications in technology.

Early proof-of-concept for the specific binding with imprinting polymerization came from Mosbach’s group [2,3]. One of the earliest applications that implemented molecularly imprinted polymers (MIPs) was the separation of chiral compounds using chiral solid phases in column chromatography [4,5]. At this point, MIPs are used in many different applications. Broadly, they can be grouped into two categories: Detection and sensing for a variety of compounds, from contaminants to proteins in cells [6,7,8,9,10,11,12,13,14,15,16,17,18] and extraction and purifications of compounds from environmental and biological samples [19,20,21,22,23,24,25].

The crosslinking density of a material determines its physical properties, such as the porosity of the material. In imprinting polymerizations, the porosity determines access to internal binding sites and thus the capacity of the imprinted material. The aim of this work is to analyze the effect of the commonly used crosslinking density in imprinting polymerization for a variety of applications. This will be accomplished by selecting current examples of imprinting polymerization and correlating the details of their syntheses with MIP capacity and polymer science data. This will not be a comprehensive review of imprinting polymerization. In fact, only a small number of studies of the vast imprinting polymerization literature will be used.

## 2. Common Syntheses for Imprinting Polymerizations

Imprinting polymerization generally uses a similar synthesis: A “functional monomer” is selected that is effective in binding the template, the “structural monomer”, which is the crosslinker, is chosen to match the polarity needed for the reaction and possibly also to bind to the template. A solution with the template and monomers is given time to bind to each other, then the initiator is added to the mixture and the polymer is formed. After isolating the polymer, the template is removed [1]. This results in specific binding sites that allow for the specific binding that differentiates imprinted polymers from non-imprinted resins [1,2,3,4,5,6,7,8,9,10,11,12,13,14,15,16,17,18,19,20,21,22,23,24,25,26].

Most commonly, imprinting polymerization is based on non-covalent forces, but covalent and semi-covalent imprinting has also been reported [27]. There are variations in where the imprinting occurs (bulk imprinting or surface imprinting [28]), as well as what materials are used (polymeric materials, inorganic materials [29] or hybrid materials [30,31]). In this work, the focus is on either bulk or surface imprinting in polymeric materials.

Looking at bulk imprinting of polymeric materials in more detail, the ratio between the template, functional monomer, and crosslinker is important [32]. The amount of functional monomer is directly related to the amount of template since there has to be sufficient functional monomer to interact with all of the template molecules. The crosslinker then fixes the three-dimensional structure that binds the template most effectively. An effective ratio between template:functional monomer:crosslinker has been identified as 1:4:20 [32]. This has been used in the following syntheses as the starting point for optimization of the system and the application in question [33].

Surface imprinting was developed due to two common problems that were found with bulk imprinting, the difficulty to remove all templates after MIP synthesis, and the difficulty to access internal binding sites [34]. In surface imprinting, the MIP is commonly prepared as a coating onto a hard particle. The starting ratio of template:functional monomer:crosslinker is also 1:4:20 [34].

## 3. The Effect of Porogen and Crosslinking on Imprinted Materials

In this work, specifically the ratio between the functional monomer and crosslinker is highlighted since that determines the physical properties of the resulting MIP. That ratio also determines the number of accessible binding sites. Table 1 lists the ratio and the total capacity for a variety of examples in recent literature. A large majority is based on the 1:5 ratio described in the preceding section.

Only studies that report the total capacity of their MIPs and the monomer:crosslinker ratio were selected for Table 1. The cited studies use templates as small as metal ions to as big as peptides and any size in between.

It is common to use porogens to increase the surface area and with that the capacity of the imprinted polymers [42,43,44,61,62,63,64,65]. Most porogens are solvents or solvent mixtures. The solubility of the template, monomer(s), and crosslinker is one of the major factors determining the surface area [44,63,65]. Using a solvent or co-solvent that is a non-solvent can lead to phase separation. If the phase separation leads to precipitation of the complex or the polymer, that generally leads to reduced surface area [42,44,63]. If the non-solvent creates an emulsion, that can lead to cracks or pores, which often increase the surface area [42]. An effective way to increase the surface area is to use a solid porogen, usually a salt particle that can later be dissolved and washed out [61,62]. Insoluble polymers have been reported as porogens, as well [61].

The properties of the crosslinker also make a difference in the surface area, pore size, and binding capacity of the resulting polymer, as well as the structure of the prepolymerization complex [36,42,44,66,67,68,69,70,71,72,73,74]. The intermolecular forces between the template, monomer, crosslinker, and solvent drive the formation of the prepolymerization complex, as was confirmed by several computational studies [66,67,68,69,70,71,73,74]. For stronger binding, it may require additional binding events occurring during oligomer formation, depending on the exact system [75]. The strongest binding to the template occurs when the intermolecular forces match between all compounds in the mixture. When properties such as polarity differ, phase separation can occur, especially with compounds that easily crystallize, such as methyl methacrylate [70,71]. As with porogens, that can cause phase separation, which leads to denser structures and less surface area. Functional monomers and crosslinkers participate in the prepolymerization according to their molar ratios and matching intermolecular forces [36,42,44,66,67,68,69,70,71,72,73,74]. This often results in a variety of structurally different prepolymerization complexes, since the strength of binding with compounds of similar properties can be similar [72,73]. The structure of the crosslinker is also important in an additional way: Multifunctional crosslinkers result in fully-crosslinked materials with a lower molar ratio than difunctional crosslinkers, since one molecule can connect more than two polymer chains [36,37,42,56,58,59,72,76].

Some studies optimize for selectivity, enantioselectivity or detection limit (in the case of sensors). All of these factors will introduce variability in the total capacity value. And yet, there is an interesting trend in the data shown in Table 1: The capacity for MIPs made by bulk polymerization and surface polymerization are similar, even most hollow MIPs fall into the same capacity range. Both surface imprinting and hollow particle MIPs were developed to allow for better access to the imprinted site and with that for increased capacity. And yet, that is not seen in the data. This suggests that most binding in all cases occurs on the surface.

There are four studies with an unusual high capacity in this selection. An’s group used polymers grafted onto a particle surface, then crosslinks the graft polymers [50]. That results in much larger pore sizes and pore volume for easier access to the pores, especially for metal ion templates that are comparatively small. Sarbu’s group used emulsion polymerization to prepare hollow particles that are much larger than most, in the range of 10s of micrometers, and thus allow easy access to a large number of imprinting sites on the surface [53]. Mishra’s [57] and Mueller’s group [60] used a much lower amount of crosslinker than the other studies. In fact, Mueller’s group achieves a very high capacity with simple bulk polymerization.

In that study, the low crosslinker concentration was chosen based on data from polymer science research. In polymer science, crosslinking is used to make a material insoluble and reduce the amount of swelling [77]. Studies with a variety of different polymer systems show that the “gel point”, i.e., the point a polymeric material becomes insoluble, can be reached with a few percent of crosslinkers compared to the monomer. The gel point is thought of as the point where all polymer chains are connected into a large network by the crosslinking molecules. At that point, the whole polymer system is one large molecule, and thus too big to be soluble. Theoretical calculations confirm that only 5 in 100 to as low as 5 in 1000 repeating units in a polymer chain have to be connected to form one big network and thus a solid material [77]. In addition, a crosslinking density of 0.1 to 1% is sufficient to encapsulate gas into a polymeric material [78].

When more crosslinkers than monomers are used, each repeating unit of a polymer chain is connected to its neighbors as well as to a repeating unit of a different polymer chain. That allows for minimal free volume between each polymer chain, likely with a lot of interspersed crystalline regions. That means that only imprinting sites on the surface are accessible for binding, and trapped templates will not be able to be removed.

Several of the cited studies use the solvent or gas as porogens to increase the free volume and pore size to allow for access of internal imprinted sites. Crosslinking, though, is known to exclude the solvent from the polymer. There are many examples where crosslinking of liquid crystalline phases leads to crystalline structures [75,79]. Another example that demonstrates how effectively the solvent is excluded during crosslinking is the shrinking of dental composites used for filling cavities [80]. There is a lot of research with the goal of reducing shrinkage in that field, since a filling that is too small for its cavity is ineffective.

Another study that illustrates the exclusion of solvent during crosslinking measures the amount of swelling with varying crosslinking densities [81]. Swelling of a material is the result of the free movement of solvent into the polymeric material. Only 10% of crosslinker was enough to stop most of the swelling.

This demonstrates another problem that internal imprinted sites have in an MIP: For a template to be able to reach the site, there has to be a continuous channel to that site, as well as a flow of solvent with the template to be able to move into the site and rebind. Especially with water as the solvent, the amount of water around a solute molecule has to be large for an aqueous solution to be free-flowing [82]. Water has shown to be very viscous due to its extensive hydrogen bonding, and around hydrophilic compounds water can be strongly bound or even crystalline [82].

Which brings up another point: The kinetics of reaching binding sites that are on the surface vs. inside a particle. Templates that bind to surface sites can bind quickly, since the binding sites are readily accessible. Templates that bind to internal sites have to move through a viscous solvent in likely bent channels to reach the binding sites. Therefore, the kinetics of binding to internal sites will always be slower than the kinetics of binding to surface sites. And yet, most studies using bulk imprinting report linear binding kinetics.

The combined evidence from polymer science suggests that when more crosslinkers than functional monomers are used, the inside of the particle is extremely dense and the internal binding sites will not be accessible. Essentially, bulk polymerization and surface polymerization will result in the same outcome, as the data in Table 1 also suggested. In fact, one has to go to very low crosslinking densities (0.5 to 5% of crosslinker) to create materials with accessible internal binding sites.

One major reason why a high crosslinking density has been used in imprinting polymerization is due to the fact that the imprint needs to be stable for the MIP to allow for specific rebinding [32]. This is based on measurements of binding constants. The strongest binding constants were found when the functional monomers were in optimal alignment with the template. Therefore, that alignment has to be preserved to achieve the highest selectivity, and that can only be done when there is no movement in the polymer chains anymore.

There is recent work that studies enzyme-substrate binding, which suggests that might not quite be true. Methods have been developed that can follow enzyme-substrate binding in real time. The results show that binding is a dynamic process [83]. Initially, the active site is a bit larger than the substrate. The substrate has room to move into the site easily but initially only binds weakly. After the initial binding, the surrounding amino acids move towards the template and bind strongly. In fact, more data in the field suggest currently that strong binding is only possible with this two-step, dynamic process [84].

## 4. Summary and Future Outlook

This review looks at the imprinting polymerization literature asking the question: How can the most accessible imprinted binding sites be generated in a material? The data suggest that with a high crosslinking density, only surface imprinted sites can be accessed, reducing the possible binding capacity.

There are always two major considerations when developing MIPs: The capacity and specificity needed for the application in question. The requirements for each application will be different in those two points.

The research reviewed here suggests that the lower crosslinking density can result in higher capacity for MIPs. When using external and internal imprinting sites for maximum capacity, large channels and solvent contents are needed. The disadvantage will be non-linear binding kinetics. Moreover, some applications that use MIPs require that the material will be pressure stable. Porous materials full of solvent generally do not withstand high pressure, but there are simple methods beyond increasing the crosslinking density that can be used to increase pressure stability [85].

What determines the specificity of binding is the percentage of functional monomers being part of imprinting sites and their alignment towards the template. Ideally, the percentage is 100%. However, that percentage is based on how freely the functional groups can move towards the template and bind strongly. That is why template binding is always the first step of imprinting polymerization, and only after binding the crosslinker and the initiator are introduced. Remember, though, that crosslinking polymerization will exclude the solvent (and possibly other small molecules), and with that the structure of the polymer chains, and with that any alignment, will change during polymerization [75]. Therefore, the exact structure of the imprinting sites will only survive if the binding of the template is stronger than the force that excludes the solvent. Additionally, the higher the crosslinking density, the stronger the exclusion force. Therefore, the lower crosslinking density might result in more effective binding sites. Moreover, the strongest binding is likely to be dynamic binding, where the functional repeating units within the polymer require the possibility to move.

In conclusion, this work suggests that reducing the crosslinking density might improve both the capacity, as well as the selectivity of imprinted materials. Therefore, more research into the optimal crosslinking density for imprinting polymerization is needed for many applications.

## Figures and Tables

**Table 1:** Functional monomer ratio and total capacity for MIPs for a variety of applications cited in selected recent literature.

Monomer:Crosslinker Molar Ratio	Template Crosslinker	Maximum Capacity (mg/g)	Comments	Reference
1:2.7	UO_2_^2+^ EGDMA ^2^	125	Bulk imprinting BET A^2^ 670 m^2^/g, pore vol. 1.439 mL/g, avg. pore Ø 2.2 nm ^1^ Adsorption dependent on pH, initial conc., regeneration	[35]
1:5	Cu(II) Pentaerythrol triacrylate ^3^	2.16	Bulk imprinting BET A^2^ 6.7 m^2^/g, pore vol. 0.0088 mL/g, avg. pore Ø 5.2 nm ^1^	[36]
1:4.5	Extracellular matrix peptides Pentaerythrol triacrylate ^3^	49.55	Bulk imprinting Most templates trapped	[37]
1:3, 1:5	Serotonin reuptake inhibitors EGDMA ^2^	27.3	Bulk imprinting BET A^2^ 193.8 m^2^/g, pore vol. 0.37 mL/g, pore Ø 7.7 nm ^1^	[38]
1:3, 1:4, 1:5	Sarafloxacin EGDMA ^2^	58.6	Bulk imprinting Several functional monomers More crosslinking, less capacity	[39]
1:4 to 1:20	Sialic acid EGDMA ^2^	24.7	Bulk imprinting Specialized acrylates 1:4 highest capacity	[40]
1:2.5	Sulfonylurea pesticides Divinylbenzene	1.6	Bulk imprinting BET A^2^: 409.7 m^2^/g ^1^	[41]
1:4	2-(3,4-dimethoxyphenyl)ethylamine Trimethylopropane trimethacrylate ^3^	24.5	Bulk imprinting Optimized crosslinker and porogen	[42]
1:0.38	Atrazine EGDMA ^2^	3.45	Bulk Imprinting Investigating porogen BET A^2^ 237.5 m^2^/g, pore vol. 0.0268 mL/g, pore Ø 0.57 nm ^1^	[43]
1:5	4-Hydroxy-3-nitrophenylacetic acid EGDMA ^2^	0.106	Bulk Imprinting Porogen, pore structure, and sorption investigation	[44]
1:5	Chloramphenicol EGDMA ^2^	64.3	Surface imprinting, hollow rods 1–3 μm long, Ø 50–180 nm ^1^	[45]
1:4.5	Peptide EDMA ^4^	76.9	Surface imprinting, hollow	[46]
1:1.2	Cytidine EGDMA ^2^	33.39	Surface imprinting, magnetic MIP BET A^2^: 980 m^2^/g ^1^	[47]
1:2.5, 1:5	Cd(NO_3_)_2_ EGDMA^2^	32	Membrane Less crosslinking, more adsorption Less imprinting molecule, less adsorption	[48]
1:1	Acteoside EGDMA^2^	62.83	Surface imprinting, membrane	[49]
1:1.3	Cd(NO_3_)_2_ Ethylene diamine	250.7	Surface imprinting Surface crosslinking only BET: A^2^ 192.2 m^2^/g, pore vol. 0.052 cm^3^/g, pore Ø 113 nm ^1^	[50]
1: 0.68	Sulfa-methoxasole EGDMA ^2^	20.0	Surface imprinting, magnetic MIP Computational study	[51]
1:0.44	Sulfonamides EDMA ^4^	0.559	Surface imprinting, magnetic MIP Hybrid with silicon	[52]
1:4	Pseudohepericin EDMA ^4^	450	Hollow particle Prepared by emulsion polymerization Inner Ø ca. 30 μm ^1^	[53]
1:5	Estrogens EGDMA ^2^	12.1	Hollow particle Ca. 250 nm inside Ø ^1^	[54]
1:5	Celecoxib EGDMA ^2^	43.29	Hollow particle	[55]
1:0.2	Cr(VI) Trimethylopropane trimethacrylate ^3^	66.6	Bulk imprinting BET: A^2^ 4.78 m^2^/g, pore vol. 0.00554 cm^3^/g, pore Ø 2.35 nm ^1^	[56]
1:0.0079	(S)-Naproxen EGDMA ^2^	127	Surface imprinting, magnetic MIP Enantioselectivity 4:1	[57]
1:2.5	Quinine Trimethylopropane trimethacrylate ^3^	15.38	Start with colloidal silica crystal microsphere Coat MIP on porous crystal, then remove crystal BET: A^2^ 216 m^2^/g, pore vol. 0.66 cm^3^/g, avg pore Ø 12.2 nm	[58]
1:1.05	Artimisin 3-Aminopropyltriethoxysilane	45.89	Start with polydopamine as the core Coat imprinted Si around by the sol-gel method Phase inversion, then cast as membrane	[59]
1:0.005	Cd(II) EGDMA ^2^	950	Bulk Imprinting Increased porosity by bubbling N through the reaction	[60]

^1^ A^2^: Surface area; Ø: Diameter. ^2^ Ethylene glycol dimethacrylate. ^3^ Trifunctional crosslinker. ^4^ Ethylene dimethacrylate.

## Data Availability

No new data were created or analyzed in this study. Data sharing is not applicable to this article.

## Sample Availability

No new samples were created or analyzed in this study. Sample sharing is not applicable to this article.
